# Hedgehog Buckyball: A High-Symmetry Complete Polyhedral Oligomeric Silsesquioxane (POSS)

**DOI:** 10.3390/polym8080315

**Published:** 2016-08-22

**Authors:** Yu Hu, You Wang, Hong You, Di Wang

**Affiliations:** 1 School of Materials Science and Engineering, Harbin Institute of Technology, Harbin 150001, China; 2 State Key Laboratory of Urban Water Resource and Environment, Harbin Institute of Technology, Harbin 150001, China; youhong@hit.edu.cn; 3 Material Science and Engineering College, Northeast Forestry University, Harbin 150040, China; diwang1030@126.com

**Keywords:** polyhedral oligomeric silsesquioxane (POSS), high-symmetry, fullerene-like silsesquioxane, synthesis and characterization

## Abstract

In this study, we report UV-MALDI-TOF MS evidence of a fullerene-like silsesquioxane, a high-symmetry polyhedral oligomeric silsesquioxane (POSS or SSO) formulated as R_60_-Si_60_O_90_ or T_60_ (T = RSiO_1.5_). The T_60_ preparation can be performed using a normal hydrolytic condensation of [(3-methacryloxy)propyl]trimethoxysilane (MPMS) as an example. Theoretically, four 3sp^3^ hybrid orbitals (each containing an unpaired electron) of a Si atom are generated before the bond formation. Then it bonds to another four atom electrons using the four generated hybrid orbitals which produced a stable configuration. This fullerene-like silsesquioxane should exhibit much more functionality, activity and selectivity and is easier to assemble than the double bonds in a fullerene.

The fullerene buckyball (FBB), C_60_, was named “Molecule of the Year” for 1991 by Science [1]. Since the discovery of the C_60_ buckyball, fullerene science has continued to accelerate, investigating both the basic science and its potential applications [2,3]. One investigation involves a major focus on its analogue, the Si_60_ cluster [4,5]. For Si_60_ clusters, the cages should not be very stable due to the use of three (3sp^3^) orbitals to bond to other Si atoms. Thus, the cages need some other atoms for the fourth bond whereas the C_60_ fullerene uses the second period sp^2^ orbitals along with a π bond to bond exclusively with other C atoms. Most investigations focus on endohedral Si_60_ isomers (using the fourth bond of the 3sp^3^ orbital) which are unstable [6]. To produce a stable Si_60_ configuration, Wang and Yang conducted ab initio calculations based on density functional theory on a Si_60_ fullerene-like cage passivated with F or Cl atoms [7]; however, this research is limited by a complex experimental synthesis.

In this study, we report UV-MALDI-TOF MS evidence of a fullerene-like silsesquioxane, a high-symmetry polyhedral oligomeric silsesquioxane (POSS or SSO) formulated as R_60_-Si_60_O_90_ or T_60_ (T = RSiO_1.5_) [8]. Theoretically, four 3sp^3^ hybrid orbitals (each containing an unpaired electron) of a Si atom are generated before the bond formation. Then it bonds to another four atom electrons using the four generated hybrid orbitals which produced a stable configuration. A significant difference between a FBB and T_60_ POSS is that the cage in the former is the four-bond (three 2sp^2^ hybrid orbits and one original 2p orbital) connection of each C atom on the FBB surface, while it is the three-bond (three of four 3sp^3^ hybrid orbits) connection of each Si atom on the POSS surface. The remaining bond to each silicon connects to a pendant organic group adorning the surface of the T_60_ cage, showing a hedgehog buckyball (HBB, see Figure 1). These organic groups exhibit much more functionality, activity and selectivity and are easier to assemble than the double bonds in a fullerene, which facilitates the synthesis of POSS-based materials possessing unique properties and the ability to set up applications [9,10,11,12,13,14].

The HBB T_60_ preparation can be performed using a normal hydrolytic condensation of [(3-methacryloxy)propyl]trimethoxysilane (MPMS) that we have used to prepare silsesquioxane coatings [15,16]. Hydrolysis and condensation normally give rise to smaller oligomers with a dozen or fewer monomers interconnected into rings [17]. By extending the condensation time we are able to produce more viscous products (M-POSS or MSSO) with molecular weights in the range calculated for methacryloxypropy-T_60_ (10,755 Daltons).

Generally the groups and the location of the groups are determined by FTIR and NMR (^1^H, ^13^C and ^29^Si); then all possible predicted structures are established by the molecular weights assigned from the peaks of UVMALDI-TOF MS and the general formula [8,18,19]. Figure S1 schemes the structural formula of MSSO, facilitating analysis and assignment of FTIR and ^1^H- and ^13^C-NMR spectra. The typical FTIR MSSO spectrum (Figure S2b) bands at 417–478 and 1122–1129 cm^−1^ are primarily ascribed to the stretching of O–Si–O and Si–O–Si; the obvious bands at about 1298, 1410 and 1724 cm^−1^ derive from stretches of CH=CH_2_ and C=O groups in methacrylate chains; a decrease in the intensity of the Si–OCH_3_ group band at 2938 cm^−1^ is observed relative to the lower molecular weight MSSO, evidenced together with the generation of a broad band at 3423 cm^−1^, assigned to –OH groups from Si–OH. Except for bands at 2938 and 3423 cm^−1^, these data are in good agreement with those of the MPMS spectrum in Figure S2a and give complementary information for the characterization of the structure.

The following peaks in the ^1^H-NMR spectrum (Figure S3) were assigned: 0.698 ppm (1); 1.784 ppm (2); 1.925 ppm (7); 3.520, 3.580 ppm (CH_3_–OH, Si–OH); 4.111, 4.103 ppm (3); 5.545, 6.091 ppm (5, 6); 7.280 ppm (CHCl_3_); 8.004, 8.049 ppm (HCOOCH_3_, HCOOH). The following peaks in the ^13^C-NMR spectrum (Figure S4) were assigned: 8.534 ppm (1); 17.865 ppm (7); 21.954 ppm (2); 66.121, 65.894 ppm (3); 76.674, 77.000, 77.319 ppm (CHCl_3_, CH_3_-OH, Si–OH); 125.006 ppm (5); 135.983 ppm (6); 162.785 ppm (HCOOH); 167.019 ppm (4, HCOOCH_3_). Besides ^1^H- and ^13^C-NMR spectroscopy, ^29^Si NMR spectroscopy permits quantitative measurement of the degree of condensation by the relative abundance of the T^3^ silicon nuclei, Si–(O–Si)_3_ [20,21,22,23]: −65.681 ppm is characteristic of the T^3^ species, and –56.577 ppm can be assigned to T^2^ structures, Si–(O–Si)_2_(OH), from incompletely condensed species in the product mixture; no T^0^ or T^1^ structures were present in the ^29^Si-NMR spectrum which is consistent with higher molecular weight and cyclic MSSO.

Figure 2 shows the UV-MALDI-TOF MS in the *m*/*z* = 0–11,000 Da range corresponding to MSSO molecules. Three high-symmetry complete MSSOs, T_8_, T_20_ and T_60_ (see Figure 3), were assigned (see Table 1). These predicted structures have a compliance between the experimental measurement value and the calculated molecular weight according to ion adducts (*M*_w_ + H^+^ or *M*_w_ + Na^+^ or *M*_w_ + K^+^) [24], however, there are still few differences, probably from ion adducts selected, solvent used for the measurement operation, calculations and so on. T_60_, one of three high-symmetry complete MSSOs, is a fullerene-like HBB and is denoted as MP-HBB as shown in Figure 3c. The difference between HBB and FBB (as mentioned above) provides an ideal vehicle for exciting research which will be timely, rapidly evolving, multidisciplinary and even appealing on an aesthetic level.

Additional corroborating evidence for the proposed cyclic structures can be observed in the gel permeation chromatography (GPC) chromatograms obtained for the MSSO samples. Figure 4 shows the mass distribution of the MSSOs measured by a GPC device which provides refractive index data. The distribution profiles indicate that the oligomers are formed in three successive groups with average molecular weights of 1413.8, 4538.8 and 16,260.0 (see Figure 4b3, b2 and b1, respectively) corresponding to fractions containing the T_60_, T_20_ and T_8_ cyclic compounds [25].

Further research will be conducted on this MP-HBB to separate it from multiple MSSO structures using a gel permeation column. The intensity of the MP-T_60_ present can be determined by GPC or size exclusion chromatography (SEC), and the mass of the elutant is again measured by a mass spectrometer [25,26].

## Figures and Tables

**Figure 1:**
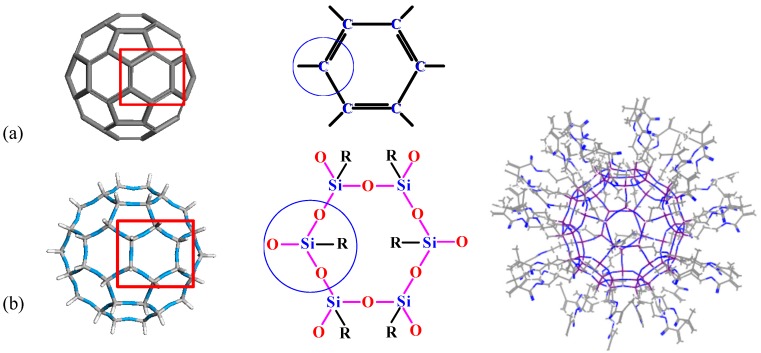
The molecular structures: (**a**) FBB, the C atom on the FBB surface connects the other three C atoms; (**b**) HBB, the Si atom connects the bridged three O atoms on the T_60_ surface and one organic group R outside the HBB surface.

**Figure 2:**
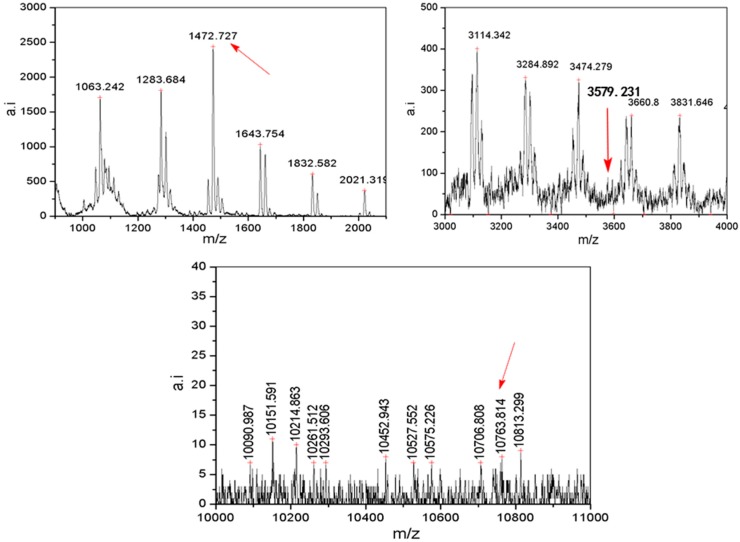
The UV-MALDI-TOF MS in the *m*/*z* = 500–11,000 Da range correspond to the MSSO oligomers.

**Figure 3:**
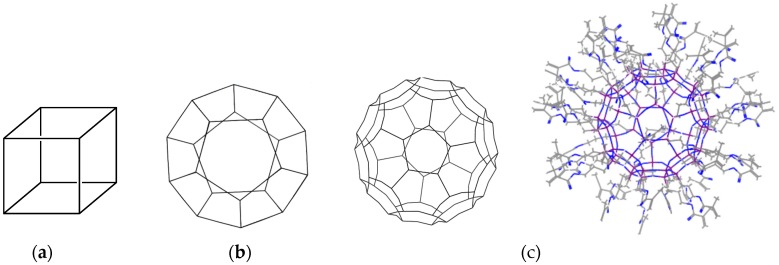
Structures of three high-symmetry complete MSSOs: (**a**) T_8_; (**b**) T_20_ and (**c**) T_60_.

**Figure 4:**
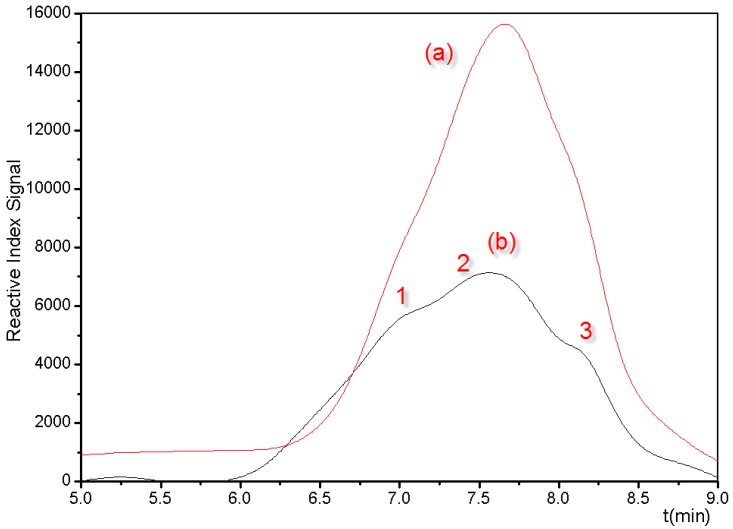
The mass distribution of the MSSO oligomers measured by a GPC device which provides refractive index data: (**a**) reaction under 70 °C for 12 days; (**b**) reaction under 35 °C for 20 days, average molecular weights of oligomers formed in three successive groups with (**b1**) 16,260.0, (**b2**) 4538.8 and (**b3**) 1413.8, corresponding to fractions containing the T_60_, T_20_ and T_8_ cyclic compounds.

**Table 1:** Three high-symmetry complete MSSOs assigned by the mass spectrum (*m*/*z* = 0–11,000 Da).

Experiment (*m*/*z*)	Assigned structure	Calculation (*m*/*z*) (+H^+^, K^+^)	Symmetry
1,472.73	R_8_Si_8_O_12_ (+K^+^)	1,472.98	O_h_
3,579.23	R_20_Si_20_O_30_ (+H^+^)	3,585.96	I_h_
10,763.81	R_60_Si_60_O_90_ (+H^+^)	10,755.88	I_h_

## Supplementary Materials

The following are available online at www.mdpi.com/2073-4360/8/8/315/s1, Figure S1: Structural formula of MSSO (numbers correspond to the assignment of ^1^H- and ^13^C-NMR peaks), Figure S2: FTIR spectrum of MSSO: (a) Original material, MPMS; (b) The hydrolytic condensation of MPMS for 10 days (40 °C), Figure S3: ^1^H-NMR spectrum (DMSO-d_6_, 25 °C) of MSSO, Figure S4: ^13^C-NMR spectrum (DMSO-d_6_, 25 °C) of MSSO.
