# Supplementary material for: Hedgehog Buckyball: A High-Symmetry Complete Polyhedral Oligomeric Silsesquioxane (POSS)
Source: Polymers (Basel). 2016 Aug 22;8(8):315. doi: 10.3390/polym8080315 (PMC6432261; doi:10.3390/polym8080315)
Supplement: Supplementary file 1 [file polymers-08-00315-s001.pdf]

Supplementary Materials: Hedgehog Buckyball: A High-Symmetry Complete Polyhedral Oligomeric Silsesquioxane (POSS)

Yu Hu, You Wang, Hong You and Di Wang

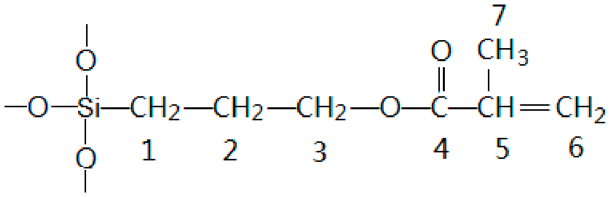

Figure S1. Structural formula of MSSO (numbers correspond to the assignment of ^{1}H- and ^{13}C-NMR peaks).

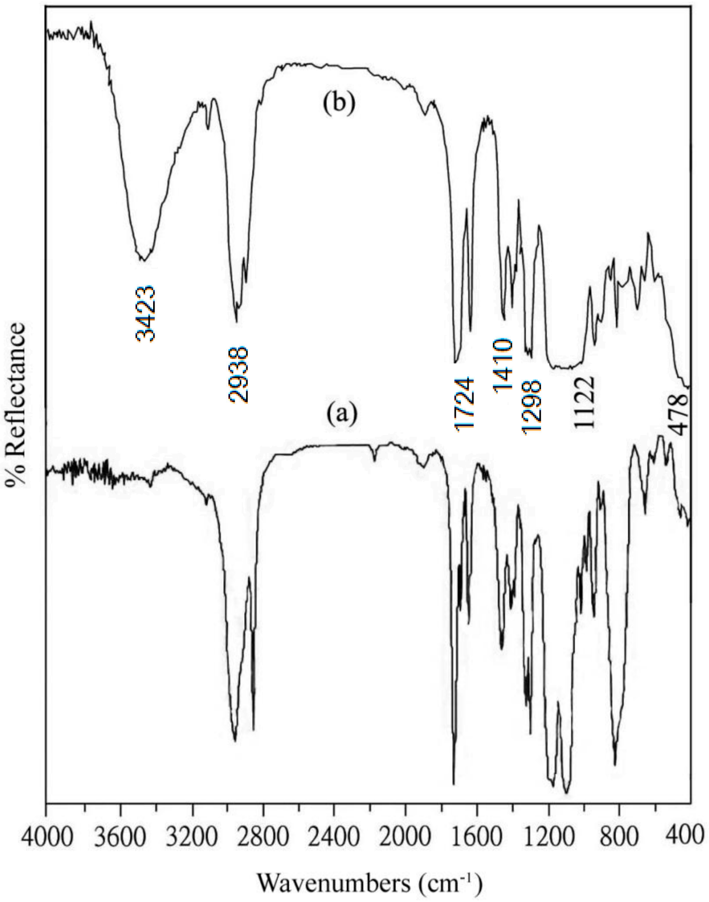

Figure S2. FTIR spectrum of MSSO: (**a**) Original material, MPMS; (**b**) The hydrolytic condensation of MPMS for 10 days (40 °C).

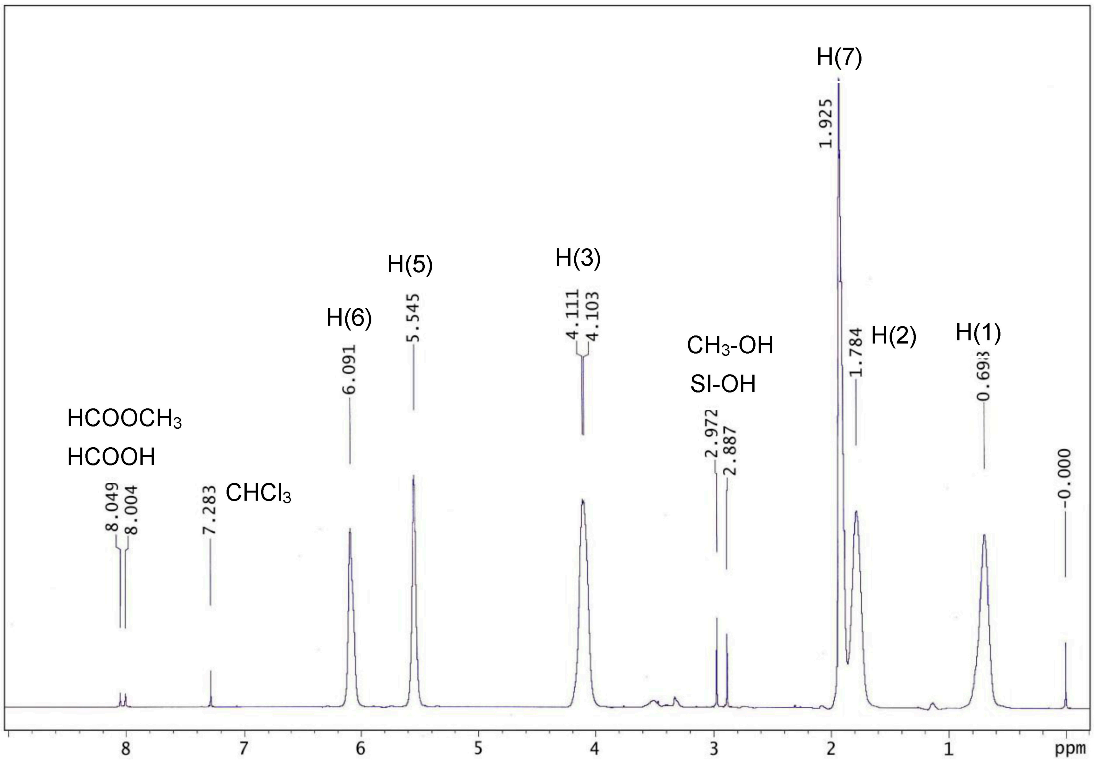

Figure S3. ^{1}H-NMR spectrum (DMSO-d_6, 25 °C) of MSSO.

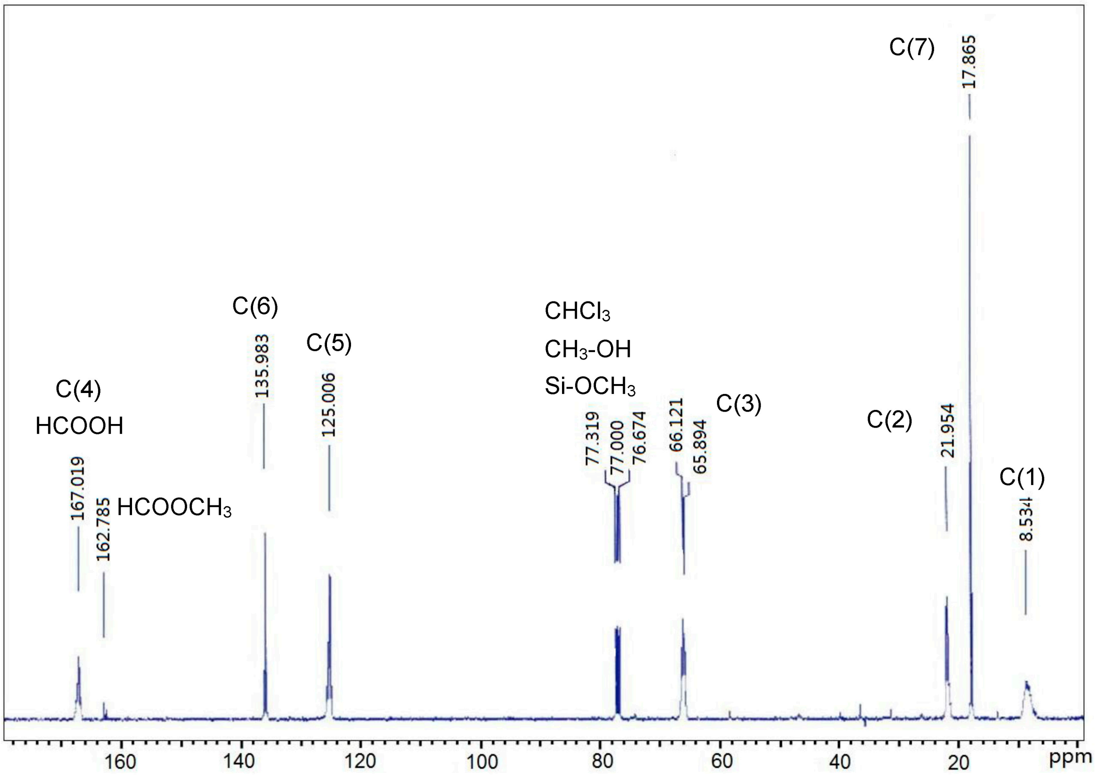

Figure S4. ^{13}C-NMR spectrum (DMSO-d6, 25 °C) of MSSO.

© 2016 by the authors; licensee MDPI, Basel, Switzerland. This article is an open access article distributed under the terms and conditions of the Creative Commons Attribution (CC-BY) license (http://creativecommons.org/licenses/by/4.0/).
